# Whole-Genome Sequencing and Comparative Genomics Analysis of the Wild Edible Mushroom (*Gomphus purpuraceus*) Provide Insights into Its Potential Food Application and Artificial Domestication

**DOI:** 10.3390/genes13091628

**Published:** 2022-09-10

**Authors:** Yangyang Geng, Shixin Zhang, Ningxian Yang, Likang Qin

**Affiliations:** 1 Key Laboratory of Plant Resource Conservation and Germplasm Innovation in Mountainous Region (Ministry of Education), College of Life Sciences/Institute of Agro-Bioengineering, Guizhou University, Hangtian West Road, Guiyang 550025, China; 2 Guizhou Institute of Walnut, 214 Fuyuan South Road, Guiyang 550005, China; 3 Guizhou Academy of Forestry, 382 Fuyuan South Road, Guiyang 550005, China; 4 School of Liquor and Food Engineering, Guizhou University, Guiyang 550025, China; 5 Guizhou Industral Technology Research Institute of Rare Edible and Medicinal Fungi Co., Ltd., 3491 Baijin Road, Guiyang 550000, China

**Keywords:** *Gomphus purpuraceus* (Iwade) Yokoyama, edible fungi, CAZymes, phylogenetic, secondary metabolisms

## Abstract

*Gomphus purpuraceus* (Iwade) Yokoyama is a species of wild fungi that grows in southwest China, considered an edible and medicinal fungus with potential commercial prospects. However, the detailed mechanisms related to the development of mycelium and the formation of the fruiting body are unclear. To obtain a comprehensive overview of genetic features, whole-genome and comparative genomics analyses of *G. purpuraceus* were performed. High-quality DNA was extracted from the mycelium, which was isolated from a fresh fruiting body of *G. purpuraceus*. The DNA sample was subjected to sequencing using Illumina and Oxford Nanopore sequencing platforms. A genome assembly totaling 40.15 Mb in 50 contigs with an N50 length of 2.06 Mb was generated, and 8705 putative predicted genes were found. Subsequently, phylogenetic analysis revealed a close evolutionary relationship between *G. purpuraceus* and *Gomphus bonarii*. Moreover, a total of 403 carbohydrate-active enzymes (CAZymes) were identified in *G. purpuraceus*, which included 147 glycoside hydrolases (GHs), 85 glycosyl transferases (GTs), 8 polysaccharide lyases (PLs), 76 carbohydrate esterases (CEs), 57 auxiliary activities (AAs) and 30 carbohydrate-binding modules (CBMs). Compared with the other 13 fungi (*Laccaria bicolor*, *Russula virescens*, *Boletus edulis*, etc.), the number and distribution of CAZymes in *G. purpuraceus* were similar to other mycorrhizal fungi. Furthermore, the optimization of culture medium for *G. purpuraceus* showed the efficient utilization of disaccharides such as sucrose and maltose. The genome of *G. purpuraceus* provides new insights into its niche, food applications and potential artificial domestication.

## 1. Introduction

Wild mushrooms are a vital source of income and nutrition for rural communities and recreational foragers. At present, a total of 2006 species from 99 countries can be consumed safely [1]. The wild edible mushroom has always been favored by people for its unique taste, flavor and nutritional value [2]. Of these wild edible fungi, some have been used as medications due to their special compounds, such as terpenes, phenolic substances, ceramides, lactones, etc., which are beneficial to the body [3,4]. The huge land areas in China provide highly diverse habitats for macrofungi, especially in northeast and southwest China and Tibet. Wu et al. considered 1020 and 692 taxa of 1662 in total to be edible and medicinal mushrooms, respectively [5]. In recent years, mushroom cultivation has become the fifth-largest agricultural industry in China. Around 100 edible mushrooms have been domesticated, of which 60 have been commercially cultivated [6]. However, many known edible or medicinal fungi have been unsuccessfully cultivated due to the uncertain ecological niches (symbiotic or saprotrophic).

**Figure 1:**
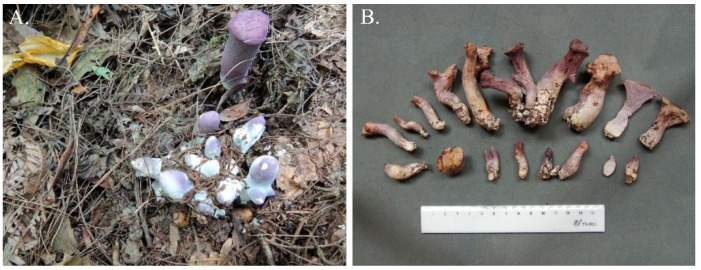
Fruiting bodies of *G. purpuraceus*. (**A**) Habitat. (**B**) Basidiomata.

*G.purpuraceus* (Iwade) Yokoyama (Figure 1A, B), an ancient and endangered species, is a kind of wild edible mushroom [7,8]. Although the fruiting bodies of the genus *Gomphus* are thought to be indigestible [9,10], *G. purpuraceus* is considered to be edible and do not cause physical discomfort, according to local consumers. Wu et al. has also classified *G. purpuraceus* as an edible mushroom without known toxicity [5]. The fruiting bodies of *G. purpuraceus* have been popular at wild mushroom markets in southwest and central China for hundreds of years, especially in Guizhou, Hubei and Sichuan Provinces, with different local names such as “matijun” (horse’s hoof) and “zituoluo”. Owing to the outstanding flavor and old health myths, some researchers have begun to focus on the extraction and functional assessment of the bioactive compounds in *G. purpuraceus*. Thus far, only two alliacane sesquiterpenes were found in the fruiting bodies of *G. purpuraceus* [11,12]. According to previous reports, terpenoids have some health-promoting properties, such as protecting the liver, detoxification, decreasing blood pressure, lowering blood fat and other medicinal functions [13,14,15]. Moreover, terpenoids have been demonstrated to effectively inhibit the DNA synthesis in carcinoma [16,17]. Therefore, *G. purpuraceus* has significant prospects for edible and medicinal development.

The genus *Gomphus* is classified as a part of the ectomycorrhizal (ECM) fungal lineage/ramaria-gautieria [18]. According to the existing ecological survey, *G. purpuraceus* fungi usually grow in stony yellow-brown soil under *Quercus* and *Pinus* trees, which indicates a possible symbiotic relationship with these trees [19]. However, there is still a lack of clear evidence of the mycorrhizal niche of *G. purpuraceus*.

As sequencing technology advances, genome sequencing has been widely applied to understand the ecological niches of wild fungi [20,21]. Comparative genomes can provide insights into the lignocellulosic decay capabilities of fungi [22,23]. In saprotrophic fungi genomes, there are abundant genes related to oxidoreductases and carbohydrate-active enzymes (CAZymes), which can help these fungi to degrade lignin (peroxidases, DyP, laccases s.s.), crystalline cellulose (GH6, GH7, AA9) and other carbohydrates (GH43, GH74) [24,25,26]. Furthermore, white-rot fungi also have abundant copies of the cellulose-binding module 1 (CBM1) to facilitate the attachment of enzymes to crystalline cellulose [27], and some oxidoreductases, especially in class II peroxidases (class II PODs), give white-rot fungi the ability to degrade lignin [20]. ECM fungi mainly obtain carbon sources from plants due to the reduction in genes encoding plant cell wall-degrading enzymes (PCWDEs), as compared to their ancestral wood decayers [28]. Nevertheless, certain ECM fungi have been reported to decompose PCWDEs existing in soil organic matter (SOM) according to transcriptome analysis [29] and the occasional discovery of fruiting bodies from *Russulaceae* on rotting wood and trunks of trees [30,31,32]. Comparative genomic analysis of ECM fungi also confirmed that certain ECM fungi have retained a unique array of PCWDEs, indicating that they possess diverse abilities to decompose lignocellulose [33,34]. To better understand fungi, the Fungal Genomics Program was held by the U.S. Department of Energy (DOE) Joint Genome Institute (JGI), which focuses on bioenergy and environmental microbiome processes [22]. Furthermore, the Mycorrhizal Genomics Initiative was put forward to reveal the symbiosis mechanisms between ECM fungi and related trees [35]. Thus, the high-quality whole genomic sequence for *G. purpuraceus* can greatly provide insights into its mycelial growth, fruiting body formation and metabolic mechanism.

Here, the whole genome of *G. purpuraceus* was de novo sequenced and assembled with a combination of Illumina and Oxford Nanopore sequencing platforms. We also performed the gene functional annotation, which provides the possibility of finding genes involved in secondary metabolism and carbohydrate metabolism. In addition, the genes encoding membrane transport proteins and CAZymes were also analyzed, and the reaction of *G. purpuraceus* mycelia in different carbon sources was also investigated, which could provide support for microbial food resources and strain propagation. On the whole, the aim of the first genome-scale assembly for *G. purpuraceus* is to develop foundational genomic and genetic resources for food application, artificial domestication and species conservation.

## 2. Materials and Methods

### 2.1. Fungal Strains

In this study, fruiting bodies of *G. purpuraceus* were collected in July 2020 from a mixed forest with scattered *Pinus massoniana* Lamb in Xiazai village, Longli County, Guizhou Province, China (26.5690° N, 106.8556° E, alt. 1149 m). The fruiting body samples were quickly transported at 4 °C to the laboratory for mycelial isolation with low glucose (2.5 g/L) modified Melin-Norkrans medium (MMN) [29]. The specimen voucher was deposited in the herbarium of the Guizhou Academy of Agriculture Sciences, with accession number GZAAS22-0001 (GAF-20071601). The strain was derived from a cylindrical fruiting body. Then, the purity of the strain was molecularly identified by ITS analysis. The pure cultures were produced by liquid culture (15 g/L fresh potato tissue, 5.0 g/L glucose, 2.0 g/L peptone, 0.5 g/L KH_2_PO_4_, 0.15 g/L MgSO_4_·7H_2_O, 2.5 mL of 10 g/L NaCl, 5.0 mL of 10 g/L CaCl_2_, 1.5 mL of 10 g/L FeCl_3_, 1 mg/L thiamine-HCl; pH 6.5) for 60 days at 25 °C, stirring with 165 rpm in darkness. Thereafter, the mycelium was collected from the liquid medium by vacuum filtration under aseptic conditions, frozen in liquid nitrogen. Finally, the samples were sent to Beijing Biomarker Technologies Co., Ltd. (Beijing, China) under dry ice conditions for DNA extraction and genome sequencing.

### 2.2. Library Construction, Sequencing and Assembly

Genome sequencing requires high-quality DNA [36]. The purity, integrity and concentration of the extracted DNA from *G. purpuraceus* were checked by Nanodrop, Qubit and 0.7% agarose gel electrophoresis. Genome survey sequences were obtained by the Illumina HiSeq X Ten sequencing platform, which can provide rapid and accurate basial information such as the genome size, heterozygosity and repeat content by the k-mer method [37]. Using the software “kmer_freq_stat” independently developed by Beijing Biomarker Technologies, the heterozygosity rate of *G. purpuraceus* genome was estimated to be 0.01% (Figure S1), which met the requirements for whole-genome construction. Then, the whole genome was sequenced by the Oxford Nanopore sequencing platform. About 15.13 Gb of raw data were generated, and the quality control of data was performed with the sequence length ≥ 2000 bp, which was subsequently corrected by Canu v1.5 [38]. Then, the corrected reads were used for genome assembly and adjusted with wtdbg2 and Racon [39]. Finally, the highly accurate genome was achieved by the correction and optimization performed with Pilon v1.14 combined with the second-generation sequencing data [40]. Genome assembly integrity was assessed through BWA and BUSCO v4.1.2 software [41,42].

### 2.3. Genomic Prediction and Genome Annotation

Through the construction and classification of the repeated sequence database, the repeated sequence rate was predicted by RepeatMasker v4.0.6 [43]. Protein coding sequences were mainly predicted by the alignment of ab initio predictions, homologous proteins and transcriptional data, and then integrated (EVM v1.1.1) and modified (PASA v2.0.2). According to the structural characteristics of non-coding RNA, the genes involved in transfer RNAs (tRNAs) were predicted by tRNAscan-SE [44]. The sequences of ribosomal RNAs (rRNAs) and other ncRNA were identified by Infernal v1.1 software based on the Rfam database [45,46]. Pseudogenes were identified by finding the termination codon or frameshift mutation using GeneWise [47]. In addition, gene clusters were predicted by antiSMASH v6.0 [48].

All predicted gene models were annotated to the functional databases, namely, Clusters of Orthologous Groups (KOG) (http://www.ncbi.nlm.nih.gov/COG, accessed on 7 August 2021), Kyoto Encyclopedia of Genes and Genomes (KEGG) (https://www.kegg.jp/, accessed on 7 August 2021), Swiss-Prot, TrEMBL (http://www.expasy.org/sprot/ and http://www.ebi.ac.uk/swissprot/, accessed on 7 August 2021) and Non-Redundant Protein Database databases (NR) (ftp://ftp.ncbi.nlm.nih.gov/blast/db/, accessed on 7 August 2021) by the similar alignment [49]. Based on annotation results from the NR database, the genes were mapped to gene ontology (GO) (http://www.ebi.ac.uk/GOA, accessed on 7 August 2021) using Blast2GO [50]. The functional annotation of the Pfam database (http://pfam.xfam.org/, accessed on 7 August 2021) used HMMER software [51]. Transporter Classification Database for membrane transport protein analyses (TCDB) (http://www.tcdb.org, accessed on 7 August 2021) and Cytochrome P450 Engineering Database (CYPED) (http://drnelson.utmem.edu/CytochromeP450.html, accessed on 7 August 2021) were also used to analyze related genes and their functional information. Pathogen–host interaction (PHI) (http://www.phi-base.org/, accessed on 7 August 2021) and the database of fungal virulence factor (DFVF) (http://sysbio.unl.edu/DFVF/, accessed on 7 August 2021) were used to evaluate the edible safety of the fruiting body of *G. purpuraceus*. Annotation of CAZymes for the *G. purpuraceus* genome was performed by HMMER based on the CAZy database (http://www.cazy.org/, accessed on 7 August 2021), which can provide insights into the way *G. purpuraceus* obtains carbon compounds, as in the study of Kohler et al. [28]. In addition, subcellular localization information is one of the key features of protein function research. Signal peptides, transmembrane proteins and secreted proteins were predicted by SignalP v4.0 (DTU Health Tech, Lyngby, Denmark), tmhmm (DTU Health Tech, Lyngby, Denmark) and EffectorP software (CSIRO, Campbell, Australia), respectively.

Mating systems are responsible for the degree of selfing/outcrossing in natural populations and impact gene flow, the accumulation of deleterious alleles and adaptability [52]. Therefore, the identification and structuring of the mating-type (MAT) genes were performed based on the genomic prediction. The MAT-A genes, including homeodomain type 1 and homeodomain type 2 MAT genes (*HD1* and *HD2* genes) in the *G. purpuraceus* genome, were identified using TBLASTN. For MAT-B genes, the pheromone receptor genes in *G. purpuraceus* were identified by the Swissprot annotation with the keyword “pheromone receptor”. The sequence length of a pheromone precursor is so short (usually 50–100 amino acid) that it could not be predicted in the normal genome annotation procedure. Transdecoder (https://transdecoder.github.io/, accessed on 7 August 2021) software with Pfam search was used to annotate the pheromone precursor by searching in the ~20 kb flanking sequence of pheromone receptor genes.

### 2.4. Comparative Genomics Analysis

The widely studied genomes of ECM edible fungi, *L. bicolor* [53], *Lactarius deliciosus* [54], *Lactarius hatsudake* [54], *R. virescens* [54], *Russula griseocarnosa* [55], *B. edulis* [33] and *Tuber melanosporum* [56], were selected for comparison with the genomic data of *G. purpuraceus* and *G. bonarii* [54]. Four saprotrophic fungi, *Lentinula edodes* [57], *Flammulina velutipes* [58], *Pleurotus ostreatus* [59] and *Morchella importuna* [60], were also added to the comparative genomics analysis, thought to have a unique ability to degrade lignin, cellulose and other phenolic compounds [24]. In addition, due to the saprotrophic properties on artificial substrates and the association with plants as an ECM symbiont, the genome sequence of *Phlebopus portentosus* was selected as the reference genome for the saprophytes and ECM fungi mentioned above [61,62]. The sequences of all selected genomic proteins were obtained from the National Center for Biotechnology Information at https://www.ncbi.nlm.nih.gov/ (accessed on 8 November 2021) and the U.S. Department of Energy (DOE) Joint Genome Institute (JGI) at https://genome.jgi.doe.gov/ (accessed on 8 November 2021) (Table S1). Because the annotation information of the selected partial genome is not available, the required information is annotated through the relevant public database in this paper.

Based on the protein sequences of *G. purpuraceus* predicted by the OrthoMCL-v2.0.3 software, gene family clustering, including cluster gene, single-copy ortholog gene families and unique gene families, was performed by comparison with selected fungi. The single-copy ortholog genes in each genome were aligned by MAFFT v7.205 with the default parameters. Every gene family was used to build the Hidden Markov Model by HMMER v3.0, and the genes with the highest scores were identified as homologous genes [63]. The conserved region was selected to construct phylogenetic trees by removing poorly aligned regions using phyML software (Microsoft Corporation, Redmond, WA, USA).

### 2.5. Optimization of Culture Medium for G. purpuraceus

The medium was optimized based on the analysis of the CAZyme genes involved in the α-amylase family and the starch and sucrose metabolism pathway (ko00500) for suitable carbon source selection. The solid medium was adopted with 2.0 g/L peptone, 0.2 g/L NH_4_Cl, 0.5 g/L KH_2_PO_4_, 0.15 g/L MgSO_4_·7H_2_O, 2.5 mL of 10 g/L NaCl, 5.0 mL of 10 g/L CaCl_2_, 1.5 mL of 10 g/L FeCl_3_ and 18 g/L agar powder. All optimization experiments were carried out in 7 cm diameter Petri dishes added with 10 g/L of different carbon sources. A 6 mm holepunch was used to pick out the culture for inoculation. Each medium formulation had 30 plates. All samples were cultivated at 25 °C in the dark for 60 days, and the measurement of colony diameters was conducted by the cross-over method [64]. The standard error was calculated by EXCEL 2016. The statistical analysis was performed using SPSS 19.0 software (SPSS Inc., Chicago, IL, USA).

## 3. Results

### 3.1. Analysis of the Genome Assembly and Gene Prediction

The analysis of the assembly scaffold and genome of *G. purpuraceus* is shown in Table 1. A total of 15,126,272,717 bp raw data were generated by the Nanopore sequencing platforms, while 13,941,417,498 bp clean reads passed the quality control check. After genome assembly, correction and optimization, a total sequence length of 40,153,731 bp was assembled into 50 contigs with an N50 length of 2,068,469 bp. The maximum contig length was 3,972,391 bp. The GC content of the assembled contig was 47.74%. The genome assembly integrity of the mapped rate and complete BUSCO was 76.84% and 252 (86.90%), respectively. The predicted result of the total repeated sequence length was 15,467,010 bp, covering 38.52% of the genomic length. Based on the alignment and integration of ab initio predictions, homologous proteins and transcriptional data, the *G. purpuraceus* genome includes 8705 coding protein genes, and the average length of protein-coding genes was 2125.01 bp. In addition, the non-coding RNA, including 11 rRNA (family number 4), 78 tRNA (family number 46) and 18 other ncRNA (family number 10), was predicted, and a total of 119 pseudogenes were found in the *G. purpuraceus* genome by GeneWise (https://www.ebi.ac.uk/Tools/psa/genewise/, accessed on 7 August 2021).

### 3.2. Gene General Annotation

**Figure 2:**
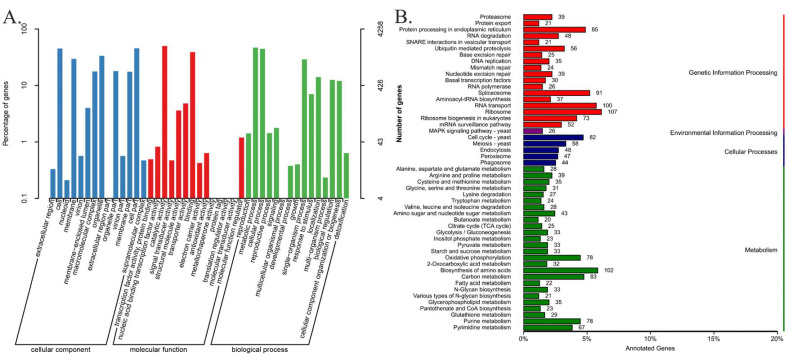
The mainly functional annotation of the *G. purpuraceus* genome. (**A**) Gene Ontology (GO) functional annotation; (**B**) Kyoto Encyclopedia of Genes and Genomes (KEGG) functional annotation.

Genome functional annotations of the 8705 predicted protein-coding genes using the public databases of GO, KEGG, KOG, Pfam, SwissProt, TrEMBL and NR are shown in Table S2. The 4258 *G. purpuraceus* protein-coding genes were annotated in the GO database, accounting for only 48.91% of the total predicted coding genes, which were divided into three major classes and 43 functional groups (Figure 2A). Among them, 14, 14 and 15 subclasses exist in the cellular component, molecular function and biological process, respectively (Table S3). A total of 3066 genes had matches in the KEGG database, which involved 109 KEGG pathways. The classification chart of the KEGG pathways is shown in Table S4. Among the 109 KEGG pathways, ribosome (107 genes), biosynthesis of amino acids (102 genes) and RNA transport (100 genes) have more abundant gene numbers than other pathways (Figure 2B).

In addition, there are 4797 genes annotated in the KOG database, shown in Table S5. The protein functions are mainly concentrated in the general function prediction only (800 genes), post-translation modification, protein turnover, chaperones (532 genes), signal transduction mechanisms (405 genes) and other aspects. The numbers of annotated genes in the Pfam, Swissprot and TrEMBL databases were 6145, 5231 and 7971, accounting for 70.59%, 60.09% and 91.57%, respectively. The largest number of genes was annotated by the NR database (94.18%), including 8198 genes (Table S6). Overall, 8212 genes were successfully annotated in the database mentioned above, accounting for 94.34% of the 8705 predicted protein-coding genes.

In the genome of *G. purpuraceus*, the two MAT-A-related genes on the contig00001 were found by BLAST homology search (Table S7). The open reading frames (ORFs) of two genes (EVM0008346.1 and EVM0002788.1) are in opposite directions, and the interval between them is 138 bp. The protein domain of the two genes was the homeobox domain. Meanwhile, HD1 (2269 bp) consists of 436 amino acids, containing three exons. HD2 (encoding 811 amino acids) has a physical length of 2486 bp on the genome, containing two exons. The mitochondrial intermediate peptidase (762 amino acids) was found to be located in close proximity with the two MAT-A-related genes on the same contig00001, which is much like that of other *Agaricomycetes*. For MAT-B genes, two potential pheromone receptor genes (EVM0003467.2 and EVM0007013.3) were identified and clustered on the contig00006. Like in other Agaricomycetes, the two genes are homologous to the STE3 gene (pheromone, a factor receptor). For pheromone genes, we searched the ~20 kb flanking region of pheromone receptor genes, but found no pheromone precursor genes in the proximity of these receptors.

### 3.3. Gene Special Annotation

The summary statistics of gene special annotation in CAZymes, P450, TCDB, PHI and DFVF are shown in Table S8.

**Figure 3:**
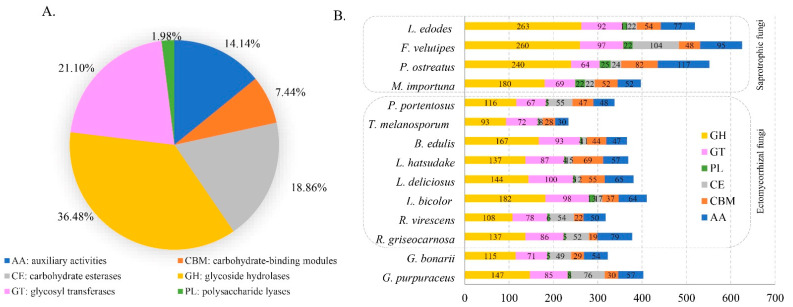
Distribution and number of carbohydrate-active enzyme genes (CAZymes) genes in *G. purpuraceus* and other 13 fungi. (**A**) Distribution of CAZymes in *G. purpuraceus*. (**B**) Comparison of CAZymes in the 14 fungi.

CAZymes of the genome can provide an insight into the metabolism of complex carbohydrates in the studied fungus. The genome of *G. purpuraceus* was compared with the CAZy database to study the CAZymes of the strain. In this database, 376 candidate CAZyme genes were identified (Figure 3A). This included 147 glycoside hydrolases (GHs, 36.47%), 85 glycosyl transferases (GTs, 21.09%), 8 polysaccharide lyases (PLs, 1.98%), 76 carbohydrate esterases (CEs, 18.85%), 57 auxiliary activities (AAs, 14.14%) and 30 carbohydrate-binding modules (CBMs, 7.44%). It is worth noting that 10 genes of *G. purpuraceus* involved in class II lignin-modifying peroxidases (AA2) were found in the CAZy database.

The antiSMASH software can accurately identify gene clusters involved in secondary metabolites of a wide range of known chemical classes. In the genome of *G. purpuraceus*, a total of 22 gene clusters were predicted by antiSMASH software. The gene cluster total length was 780,051 bp, and the gene cluster mean length was 35,456 bp. These gene clusters were distributed over 13 gene clusters. Among them, there are nine gene regions associated with terpenes, six gene regions involved in non-ribosomal peptide synthase (NRPS) or NRPS-like, five gene regions related to polyketide synthase (PKS) or PKS-like and one region associated with siderophore. In addition, one gene region was identified as both terpene and NRPS-like. The summary of predicted metabolite gene clusters is shown in Table S9.

A total of 76 genes were annotated in the TCDB database (Table S10), accounting for 0.87% of the predicted genes. About 25.56% of the predicted protein-coding genes in the *G. purpuraceus* genome were annotated in the PHI database, totaling 2225 genes (Table S11). Moreover, 1632 genes were found in the DFVF database, accounting for 18.75% of the predicted genes (Table S12).

In addition, studies have found that a class of secreted effector protein with less than 300 amino acids was related to the mycorrhizal formation [65,66,67]. The predicted results of signal peptides, transmembrane proteins, secreted proteins and effector proteins are shown in Table S13. The transmembrane proteins were found to have the highest number (1795), followed by signal peptides (673), secreted proteins (400) and effector proteins (32). 

**Figure 4:**
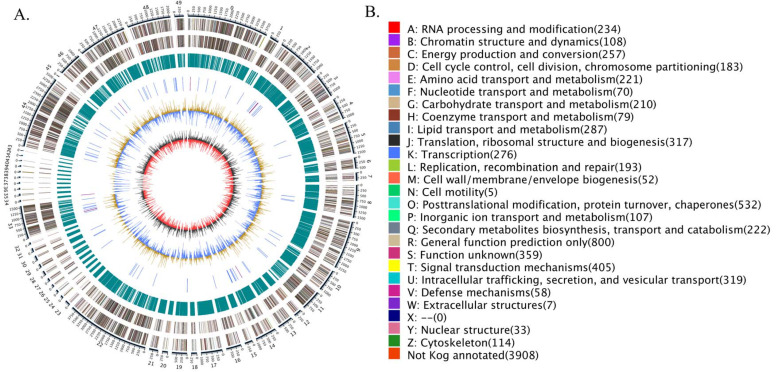
The *G. purpuraceus* circular genome diagram. (**A**) The circular genome diagram. (**B**) The KOG functional classification in circular diagram.

Finally, a detailed circular genome diagram of *G. purpuraceus* is displayed in Figure 4. The outermost circle is labeled with the size of the genome, and each scale is 50 Kb. From the outside to the inside (Figure 4A), the second and third loops represent the characteristics of the whole genome, including the positive chain gene and negative chain gene, where different colors represent different KOG functional classifications (Figure 4B). The fourth circle indicated the repeated sequence. The fifth loop exhibited the ncRNA, including tRNA (blue part) and rRNA (purple part). The sixth circle is the GC content; the outward light yellow part indicates that the GC content in this region is higher than the average GC content in the genome. The higher the peak value, the greater the difference from the average GC content. The inward blue part is the opposite. The innermost layer is the GC skew (the specific algorithm: G−C/G+C), where the outward dark gray part indicates that the content of C in this area is lower than that of G, and the inward red part is the opposite.

### 3.4. Comparative Genomics Analysis

#### 3.4.1. Gene Family Analysis

**Figure 5:**
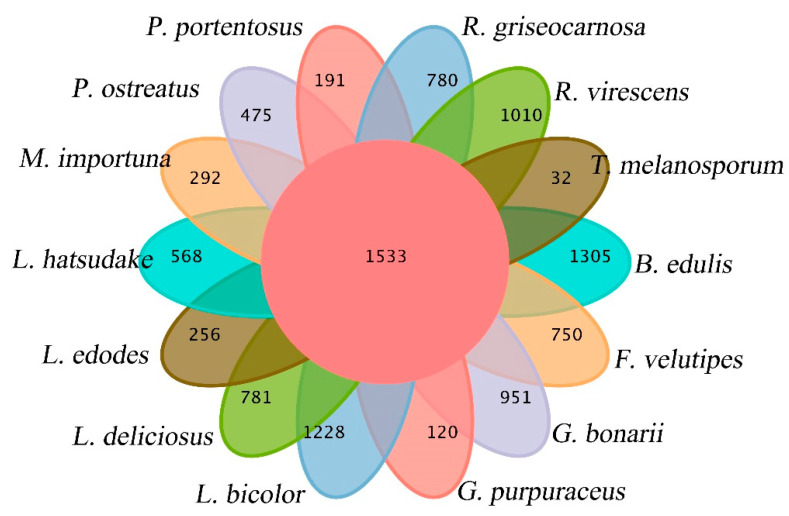
Venn diagram of orthologous gene families.

The gene families of *G. purpuraceus*, *L. bicolor*, *L. deliciosus*, *L. hatsudake*, *R. griseocarnosa*, *R. virescens*, *G. bonarii*, *B. edulis*, *T. melanosporum*, *L. edodes*, *F. velutipes*, *P. ostreatus*, *M. importuna* and *P. portentosus* were subjected to cluster analysis. The Venn diagram of common and unique gene families among 14 fungi is shown in Figure 5. The numbers of total genes, total gene families and cluster genes are listed in Table 2. A total of 22,569 gene families from 14 species were obtained by OrthoMCL. There were 6590 gene families found in the *G. purpuraceus* genome, of which 1533 were core orthologous gene families among 14 fungi genomes and 120 were unique gene families found in *G. purpuraceus* (Figure 5). Among these genomes, the number of common gene families with single-copy genes was 573.

#### 3.4.2. Phylogenetic Relationship of *G. purpuraceus*

**Figure 6:**
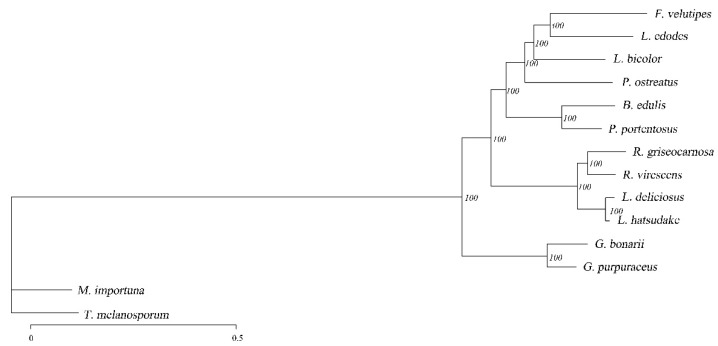
Maximum likelihood phylogenetic tree based on single-copy ortholog genes.

According to the results of 573 single-copy genes identified by gene family clustering, a phylogenetic tree was constructed, as shown in Figure 6. The value on a branch of the phylogenetic tree represents the branch reliability, and the number close to 100 represents high reliability. The bootstrap value was 100% of highly supported internal branches, which accurately reflects the availability of the datasets and evolutionary relationship of these 14 fungi. In the phylogenetic tree, most species of the same genus are clustered in the same branch, except for some saprophytes. In the NCBI taxonomy, *L. deliciosus* and *L. hatsudake*, *R. griseocarnosa* and *R. virescens* were found to belong to the genus *Lactarius* and *Russula*, which was also confirmed by phylogenetic analysis. In addition, *L. bicolor* is in the same evolutionary branch as *P. ostreatus*, and the evolutionary distance of *P. ostreatus* is greater based on the branch length. As far as the target fungus is concerned, phylogenetic analysis indicated that *G. purpuraceus* was most related to *G. bonarii*.

The phylogenetic analysis was based on different lifestyle fungi, and these different trophic types of fungi were selected for comparative genomic analysis. This provides a foundation for the functional genomics analysis of *G. purpuraceus*. 

#### 3.4.3. Comparative Analysis of CAZymes

The CAZyme composition characteristics in *G. purpuraceus* were used to compare with those of 13 other fungi, including eight ECM, four saprotrophic fungi and *Phlebopus portentosus* considered as a facultative saprotrophic fungus. The global statistics and comparisons are shown in Figure 4B and Table S14, respectively. Compared with other saprotrophic species, *G. purpuraceus* had few CAZyme protein families, especially GHs, showing that it is similar to ECM fungi such as *B. edulis*, *L. deliciosus*, *R. griseocarnosa* and *G. bonarii*. The dominant GH families are mainly involved in the degradation of plant cell wall polysaccharides, such as GH6, GH7, GH12, GH45, GH61 and AA9 (LPMOs) in the degradation of cellulose. Apart from *G. purpuraceus* (GH6, two genes; GH7, one gene) and *G. bonarii* (GH6, two genes; GH7, two genes), these two CAZyme families were completely absent from these ECM fungi genomes, showing that the ECM fungi have little ability to degrade crystalline cellulose [20]. In the AA9 CAZyme family, the number of LPMOs (auxiliary activity family 9-type) in the *G. purpuraceus* genome was much lower than that of saprotrophic fungi, as well as the other six ECM fungi, except for *L. deliciosus* and *L. hatsudake*, which oxidatively attack cellulose chains [68]. In these CAZyme families involved in the degradation of hemicellulose, such as GH3, GH10 and GH115, the *G. purpuraceus* genome also appeared to encode relatively few enzymes involved in the degradation of hemicellulose, like other ECM fungi. Compared with these ECM fungi, the CAZyme families involved in the digestion of hemicellulose were increased in the four saprotrophic fungi. In addition, the carbohydrate-binding module belonging to family 1 (CBM1) as an additional domain that promotes the absorption of cellulose was found to have three genes in the *G. purpuraceus* genome, which is indispensable for the efficiency of CBH-type catalytic domains (i.e., processive cellulases) in depolymerizing crystalline regions of cellulose [64]. Furthermore, the pectin-degrading enzymes (GH43, GH51, GH93, PL1, PL3 and PL4) in *G. purpuraceus* and other symbiotic fungi were also exhibiting a similar pattern. For the AA1 family, there may be four genes in the *G. purpuraceus* genome through the CAZy database annotation, which is widely present in fungi and is currently divided into three subfamilies, laccases, ferroxidases and laccase-like multicopper oxidases (http://www.cazy.org/AA1.html, accessed on 8 November 2021). For the CAZyme genes encoding ligninolytic enzymes, focusing on class II peroxidases (AA2), *G. purpuraceus* had a maximum of 10 genes encoding class II PODs. However, this does not prove that *G. purpuraceus* can degrade lignin since the class II PODs-encoding genes were found in a phylogenetically wide range of ECM fungi [69]. Considering the highly efficient ability of *P. portentosus* to degrade starch [70], these CAZyme families encoding α-amylase enzymes (e.g., GH13, GH70, GH77, GH57, GH119 families) were investigated. Similar to the *P. portentosus* genome (Table S14), the main α-amylase enzyme clan was mainly in GH13, while GH70, GH77, GH57 and GH119 were all absent in the selected genomes. A total of 33 genes found in the *G. purpuraceus* genome were involved in the starch and sucrose metabolism pathway.

The results of the comparative analysis of CAZymes showed that *G. purpuraceus* may have lost the capacity to completely degrade plant cell walls, though it still retains some genes for degrading PCWDEs, similar to *R. griseocarnosa* [55]. However, the abundant genes in GH 13, similar to *P. Portentosus*, suggest that *G. purpuraceus* may have a certain ability to degrade starch, which needs further confirmation.

#### 3.4.4. Comparative Analysis of Secondary Metabolisms

Fungi are a rich source of bioactive secondary metabolites, and mushroom-forming fungi are especially known for the synthesis of numerous bioactive and often cytotoxic sesquiterpenoid secondary metabolites [71]. The comparison of secondary metabolisms of *G. purpuraceus* with those of 13 other fungi was performed by genome mining and searching based on previous studies. The results are shown in Table 3. From only 14 selected fungal genomes, most basidiomycetes appear to have a greater number of terpene-involved genes than ascomycetes. However, more statistical data are required to support this conclusion in both taxa. Meanwhile, there was no obvious difference between ECM fungi and saprotrophic fungi.

In plants and fungi, terpenoids, or modified terpenes, are an important group of natural bioactive products [72,73]. For ectomycorrhizas in nature, terpenoids have been recognized as key compounds, especially sesquiterpenes. ECM fungi can promote lateral root formation by releasing sesquiterpenes [74]. In the genome of *G. purpuraceus*, there are seven gene clusters associated with terpene biosynthesis. Through checking the *G. purpuraceus* genes in the “terpenoid backbone biosynthesis (map00900)” pathway, 16 key genes were identified, shown in Table 4. Just like most fungi, terpenoid backbone biosynthesis in *G. purpuraceus* can also proceed through the mevalonate pathway (Figure S2) [75]. 

In addition, NRPSs are considered candidates for roles in fungal pathogenesis, because the products of several NRPSs have already been proven as virulence factors, such as the virulence of *Fusarium avenaceum* on potatoes, HC-toxin for *Cochliobolus carbonum* race 1 on corn and AM-toxin for *Alternaria alternata* on apple [76]. In the genome of *G. purpuraceus*, a total of 10 NRPS and NRPS-like were identified (Table 3). Through comparative analysis, the number of NPRS and NRPS-like of *G. purpuraceus* is not too much, even lower than *L. Edodes* and *P. ostreatus*, which are common edible mushrooms. Even so, the pathogenicity of *G. purpuraceus* still needs to be confirmed through a series of experiments.

### 3.5. Optimization of Culture Medium

**Figure 7:**
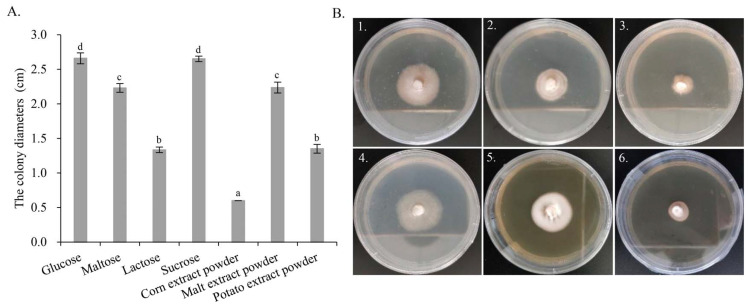
The colony diameters and morphology of *G. purpuraceus* cultured on different carbon sources. (**A**) Colony diameters. Different letters on the column indicate a significant difference (*p* < 0.01). (**B**) Morphology of *G. purpuraceus* on different carbon sources after 60 days of cultivation: (B1) glucose, (B2) maltose, (B3) lactose, (B4) sucrose, (B5) malt extract powder, (B6) potato extract powder.

From the genome annotation results, *G. purpuraceus* exhibited a limited ability to degrade lignocellulose. Comparative genomics analysis showed that the gene families associated with starch and sucrose metabolism were abundant, which is similar to *P. Portentosus*. Therefore, seven different carbon sources were further screened to find the most suitable culture medium for food fermentation and strain propagation. Measurements of the colony diameter, the growth parameter and morphology of *G. purpuraceus* are shown in Figure 7. From the colony diameter (Figure 7A), the fastest growth of *G. purpuraceus* is glucose, reaching 2.66 cm, followed by sucrose (2.65 cm), malt extract powder (2.24 cm), maltose (2.23 cm), potato extract powder (1.35 cm) and lactose (1.34 cm). It is confusing that *G. purpuraceus* cannot utilize the corn extract powder, where the colony diameter still showed the original 0.6 cm. The results showed that sucrose and maltose were efficiently utilized by *G. purpuraceus*, except for lactose. In the medium added with potato and corn extract powder containing a lot of starch, the ability of *G. purpuraceus* to utilize starch was not as efficient as *P. Portentosus* [70]. As for colony diameter, the utilization of sucrose by *G. purpuraceus* was comparable to that of glucose, and there was no significant difference between them (*p* > 0.05). The utilization of maltose or malt extract powder was second only to glucose and sucrose, with a significant difference (*p* < 0.01). In addition, the morphology of *G. purpuraceus* in malt extract powder exhibited denser mycelium and healthy growth (Figure 7B-5). The colony diameters and morphology of *G. purpuraceus* cultured on different carbon sources provided a reference for the selection of cheap carbon sources for the large-scale production of strains, such as sugarcane juice usually used in yeast fermentation.

## 4. Discussion

*G. purpuraceus* is a potential edible and medicinal wild fungus. For the purpose of artificial domestication, we sequenced the whole genome of *G. purpuraceus* and compared it with the genomes of 13 edible fungi, including white-rot, brown-rot and ECM edible fungi. Among the 14 fungal genomes, the average number of CAZymes showed a publicly recognized phenomenon: white-rot fungi were larger than brown-rot fungi, and brown-rot fungi were larger than symbiotic fungi, which may be related to the role of different ecological niches of edible fungi in the environment [20,21,22]. In the genome of *G. purpuraceus*, CAZyme protein families exhibited similarity to those of the ECM fungi, and the global numbers of GHs, GTs, PLs, CEs, CBMs and AAs of *G. purpuraceus* were less than those of typical white/brown-rot fungi. Some species of *Gomphus* are ectomycorrhizal, and the mycorrhizal system between *Gomphus floccosus* and *Abies religiosa* has been successfully synthesized under controlled conditions [10], which indicates that *G. purpuraceus* may also be an ECM fungus.

The PCWDEs are a major pool of organic carbon, so the ability to decompose PCWDEs is crucial to the artificial domestication of wild edible mushrooms. For white-rot fungi, the extracellular oxidative enzymes, particularly class II PODs, give white-rot fungi the ability to completely degrade plant cell wall components, including cellulose, hemicellulose and lignin [77]. Moreover, glycoside hydrolases (GHs) in white-rot fungi can break down crystalline cellulose. The comparative genomics analysis also showed that the numbers of GH, CE, AA and CBM family members are far greater than other niches of fungi, which provides the genetic basis for the degradation ability of white-rot fungi. In addition to white-rot fungi, brown-rot fungi are also important components of plant-degrading filamentous fungi. Due to the absence of a series of oxidative enzymes related to lignin degradation, especially in class II PODs, GH6, GH7 and family 1 carbohydrate-binding module (CBM1), brown-rot fungi only cause complete degradation of polysaccharides and partial degradation of lignin [78,79]. In brown-rot fungi, the initial decomposition of lignocellulose takes place through a nonenzymatic step including hydroxyl radicals generated by the Fenton reaction [25]. This is also demonstrated by the statistics and comparisons of CAZymes among 14 fungi. Owing to these key genes involved in the degradation of lignocellulose, many white/brown-rot mushrooms can be successfully domesticated artificially, such as *L. edodes*, *F. velutipes*, *P. ostreatus*, etc. Another large group of fungi found in nature is ECM fungi. ECM fungi mainly obtain carbon sources from tree roots by forming ectomycorrhiza [28,80]. However, obtaining carbon sources from plants does not mean that the fungi have completely lost the ability to degrade lignocellulose that accumulates in wood and SOM [29,34]. The ability of certain ECM fungi to degrade lignocellulose has long been observed in fruiting bodies found on rotting wood and trunks of trees [31,32], which was also confirmed by comparative genomic analysis [33,34]. In particular, *P. Portentosus* has been successfully domesticated in China and Thailand, despite being considered an ECM fungus. Therefore, the artificial domestication of wild edible fungi should not be restricted by the niches of ectomycorrhizal fungi. 

Considering the artificial domestication of *G. purpuraceus*, genes encoding CAZymes were analyzed. In the *G. purpuraceus* genome, GH6 (two genes) and GH7 (one gene) were closely related to the degradation of plant cell wall polysaccharides, which were completely absent from eight other ECM fungi. Therefore, the results suggested that *G. purpuraceus* fungi have a certain ability to degrade crystalline cellulose. For LPMOs (AA9) involved in attacking cellulose chains, there were only two genes in the genome of *G. purpuraceus*, even less than some ECM fungi, such as *R. griseocarnosa*, *L. bicolor* and *B. edulis*. Meanwhile, three genes associated with CBM1 that promote the absorption of cellulose were found. In addition, 10 genes encoding class II PODs were found, which are the most abundant in the 14 fungi. Furthermore, laccases (multicopper oxidases) are widely distributed in plants, fungi, bacteria and insects, which are important to biosynthesis and lignin degradation, morphogenesis and pigment biosynthesis, among others [81]. For *G. purpuraceus*, only four genes existed in the AA1 family, which was lower than ECM and saprotrophic fungi, except for *G. bonarii*, *M. importuna* and *F. velutipes*. The few genes encoding laccase reduced the ability of *G. purpuraceus* to utilize lignocellulose existing in rotting wood or SOM. On the whole, the discovery of key genes encoding CAZymes indicated that *G. purpuraceus* may have a certain ability to degrade lignocellulose. However, whether these key genes involved in lignocellulosic degradation can meet the demand for the carbon source in the life cycle of *G. purpuraceus* must be further explored. Additionally, genes involved in the α-amylase family and starch and sucrose metabolism pathway were abundant in the *G. purpuraceus* genome, just like *P. portentosus*. To preliminarily investigate the ability to utilize different carbon sources, especially in starch, the optimization of culture medium for *G. purpuraceus* was performed. The results showed that starch from potato and corn extract powder could not be efficiently utilized by *G. purpuraceus*. The maximum colony diameter in sucrose indicated that *G. purpuraceus* could efficiently utilize sucrose, which was confirmed by 13 potential genes encoding glycosidase. According to the description of malt powder product ingredients, malt extract powder contains a variety of carbohydrates, proteins, peptides, amino acids, purines and pyrimidines, as well as various vitamins, etc. Considering the addition of peptone in the basial formulation, the growth of *G. purpuraceus* requires the direct supply of purines, pyrimidines and vitamins, in addition to carbon sources, rather than obtaining them from complex substances. Therefore, we posit that the morphology of *G. purpuraceus* would exhibit healthy growth in the medium containing sucrose/maltose, peptides, amino acids, purines, pyrimidines and various vitamins. Of course, the hypothesis needs verification in further research, and the formulation still must be further investigated.

In addition to focusing on the ability of *G. purpuraceus* to acquire carbon sources, the mating type genes that are crucial in the sexual development of mushroom-forming have also been preliminarily identified. Mating is governed by mating genes located at distinct loci. In most basidiomycetes, there are two complex MATs (MAT-A and B) that control sexual compatibility in the monokaryons and regulate the maintenance of the dikaryotic state [53]. The typical MAT-A locus contains one or more pairs of genes for two types of homeodomain transcription factors, which regulate clamp-cell formation and conjugate nuclear division. Meanwhile, the MAT-B regulates nuclear migration and clamp-cell fusion [82]. Generally, the homeodomain-encoding genes and the flanking genes in the MAT-A locus are highly conserved in most mushroom-forming fungi, while the MAT-Bs of different mushrooms vary in the genomic organization. Overall, additional research is needed to elucidate the fruiting body formation of *G. purpuraceus* by MAT genes. 

## 5. Conclusions

In summary, we performed Nanopore sequencing and de novo assembly of the *G. purpuraceus* genome and provided a 40.15 Mb genome sequence consisting of 50 scaffolds with an N50 length of 2.06 Mb. The *G. purpuraceus* genome allowed us to predict the gene functions and study the biosynthesis of active compounds. The comparative genomics analysis of gene family, CAZymes and secondary metabolism may enhance our understanding of the survival mechanisms and saprotrophic capacity of *G. purpuraceus*. Moreover, optimization of the culture medium for *G. purpuraceus* also provided a foundation for food fermentation and strain propagation. The elucidation of the *G. purpuraceus* genome in this study provides the foundational information to further study the niche, biosynthesis of pharmacologically active compounds and functional food applications of *G. purpuraceus* by molecular biology techniques.

## Figures and Tables

**Table 1:** The analysis of the gene assembly and genome of *G. purpuraceus*.

Parameter	Value
*Gene assembly*	
Contigs number	50
GC content (%)	47.74
N50 (bp)	2,068,469
N90 (bp)	391,632
*Gene prediction and analysis*	
Genome size (bp)	40,153,731
Number of protein-coding genes	8705
Gene total length (bp)	18,498,255
Gene average length (bp)	2125.01
CDSs total length (bp)	12,578,619
CDSs number	62,798
Exon length (bp)	14,695,320
Intron length (bp)	3,802,935
Repeated sequence percentage (%)	38.52
Non-coding RNA number	107
rRNA number	11
tRNA number	78
Pseudo gene number	119

**Table 2:** Statistical results of the gene families.

Species	Total Gene Number	Total Gene Family Number	Cluster Gene Number
*L. edodes*	9804	6136	7776
*F. velutipes*	14,460	7393	12,308
*P. ostreatus*	11,693	7333	10,130
*M. importuna*	11,971	6237	7649
*P. portentosus*	9899	6537	9104
*T. melanosporum*	7496	5503	5812
*B. edulis*	18,718	8153	15,426
*L. hatsudake*	16,831	8553	13,716
*L. deliciosus*	18,193	8772	14,583
*L. bicolor*	18,215	7721	13,863
*R. virescens*	20,499	7121	17,450
*R. griseocarnosa*	17,754	6593	15,618
*G. bonarii*	29,452	6309	25,493
*G. purpuraceus*	8705	6590	7875

**Table 3:** Comparison of secondary metabolism of *G. purpuraceus* with 13 other fungi.

Secondary Metabolisms	Terpenes	NRPS and NRPS-like	PKS and PKS-like	DMAT	Siderophore	Hybrid	Other	Total
*G. purpuraceus*	9	6	5		1		1	22
*G. bonarii*	14	3	2					19
*R. griseocarnosa*	8	1			1			10
*R. virescens*	9	1			2			12
*L. bicolor*	9	4	3	1				17
*L. deliciosus*	18	2	2					22
*L. hatsudake*	14	2	2					18
*B. edulis*	8	9	2			1		20
*T. melanosporum*	0	4	4					8
*P. portentosus*	7	7	3				3	20
*M. importuna*	1	6	3					10
*P. ostreatus*	10	9	2					21
*F. velutipes*	12	3	1		2		2	20
*L. edodes*	7	10	5					22

Abbreviations: NRPS, nonribosomal peptides; PKS, polyketides; DMAT, dimethylallyltryptophan synthase.

**Table 4:** Putative genes involved in terpenoid backbone biosynthesis.

Symbol and Definition	EC No.	KO Term	Gene ID
ACAT, acetyl-CoA C-acetyltransferase	EC:2.3.1.9	K00626	EVM0002057.1
HMGCS, hydroxymethylglutaryl-CoA synthase	EC:2.3.3.10	K01641	EVM0008435.1
HMGCR, hydroxymethylglutaryl-CoA reductase (NADPH)	EC:1.1.1.34	K00021	EVM0004540.1
mvaK2, phosphomevalonate kinase	EC:2.7.4.2	K00938	EVM0000866.1
mvaD, diphosphomevalonate decarboxylase	EC:4.1.1.33	K01597	EVM0000734.1
IDI, isopentenyl-diphosphate Delta-isomerase	EC:5.3.3.2	K01823	EVM0005424.1
FDPS, farnesyl diphosphate synthase	EC:2.5.1.1 2.5.1.10	K00787	EVM0002481.1
GGPS1, geranylgeranyl diphosphate synthase, type III	EC:2.5.1.1 2.5.1.10 2.5.1.29	K00804	EVM0001739.1
PCYOX1, prenylcysteine oxidase/farnesylcysteine lyase	EC:1.8.3.5 1.8.3.6	K05906	EVM0005178.1
ICMT, protein-S-isoprenylcysteine O-methyltransferase	EC:2.1.1.100	K00587	EVM0004818.1
STE24, STE24 endopeptidase	EC:3.4.24.84	K06013	EVM0003020.1
RCE1, prenyl protein peptidase	EC:3.4.22.-	K08658	EVM0006143.2
FNTB, protein farnesyltransferase subunit β	EC:2.5.1.58	K05954	EVM0000741.3
FNTA, protein farnesyltransferase/geranylgeranyltransferase type-1 subunit α	EC:2.5.1.58 2.5.1.59	K05955	EVM0000039.3
DHDDS, ditrans, polycis-polyprenyl diphosphate synthase	EC:2.5.1.87	K11778	EVM0004045.1
hexPS, hexaprenyl-diphosphate synthase	EC:2.5.1.82 2.5.1.83	K05355	EVM0005717.4

## Data Availability

The original contributions presented in the study are included in the article/Supplementary Materials. The datasets used and/or analysed during the current study are available from the corresponding author. All the sequences were submitted on NCBI. For raw data, the accession numbers are SRR18321074. This Whole Genome Shotgun project has been deposited into DDBJ/ENA/GenBank under the accession JALBUN000000000. The bioproject accession is PRJNA815479, and the biosample accession is SAMN26589593.

## Supplementary Materials

The following supporting information can be downloaded at: https://www.mdpi.com/article/10.3390/genes13091628/s1, Figure S1: Kmer distribution of *G. purpuraceus* genome; Figure S2: The genes involved in the terpenoid backbone biosynthesis pathway identified from the *G. purpuraceus* genome (shown in blue); Table S1: Fungi and the origin of their genomes used in the study; Table S2: Summary statistics of functional annotation of predicted genes in public databases; Table S3: Gene Ontology (GO) functional classification of the *G. purpuraceus* genome; Table S4: Kyoto Encyclopedia of Genes and Genomes (KEGG) functional annotation of the *G. purpuraceus* genome; Table S5: Clusters of Orthologous Groups (KOG) functional classification of the *G. purpuraceus* genome; Table S6: Pfam, Swissprot and TrEMBL and NR functional annotation of the *G. purpuraceus* genome; Table S7: Homologue genes identified in the mating-type loci of *G. purpuraceus*; Table S8: Summary statistics of functional annotation of predicted genes in public databases; Table S9: The secondary metabolites gene clusters in *G. purpuraceus*; Table S10: TCDB annotation of the *G. purpuraceus* genome; Table S11: PHI annotation of the *G. purpuraceus* genome; Table S12: DFVF annotation of the *G. purpuraceus* genome; Table S13: The subcellular localization information of the *G. purpuraceus* genome; Table S14: Distribution of CAZyme in *G. purpuraceus* and 13 other fungi.
