# Supplementary figures and images for: Whole-Genome Sequencing and Comparative Genomics Analysis of the Wild Edible Mushroom (Gomphus purpuraceus) Provide Insights into Its Potential Food Application and Artificial Domestication
Source: Genes (Basel). 2022 Sep 10;13(9):1628. doi: 10.3390/genes13091628 (PMC9498453; doi:10.3390/genes13091628)

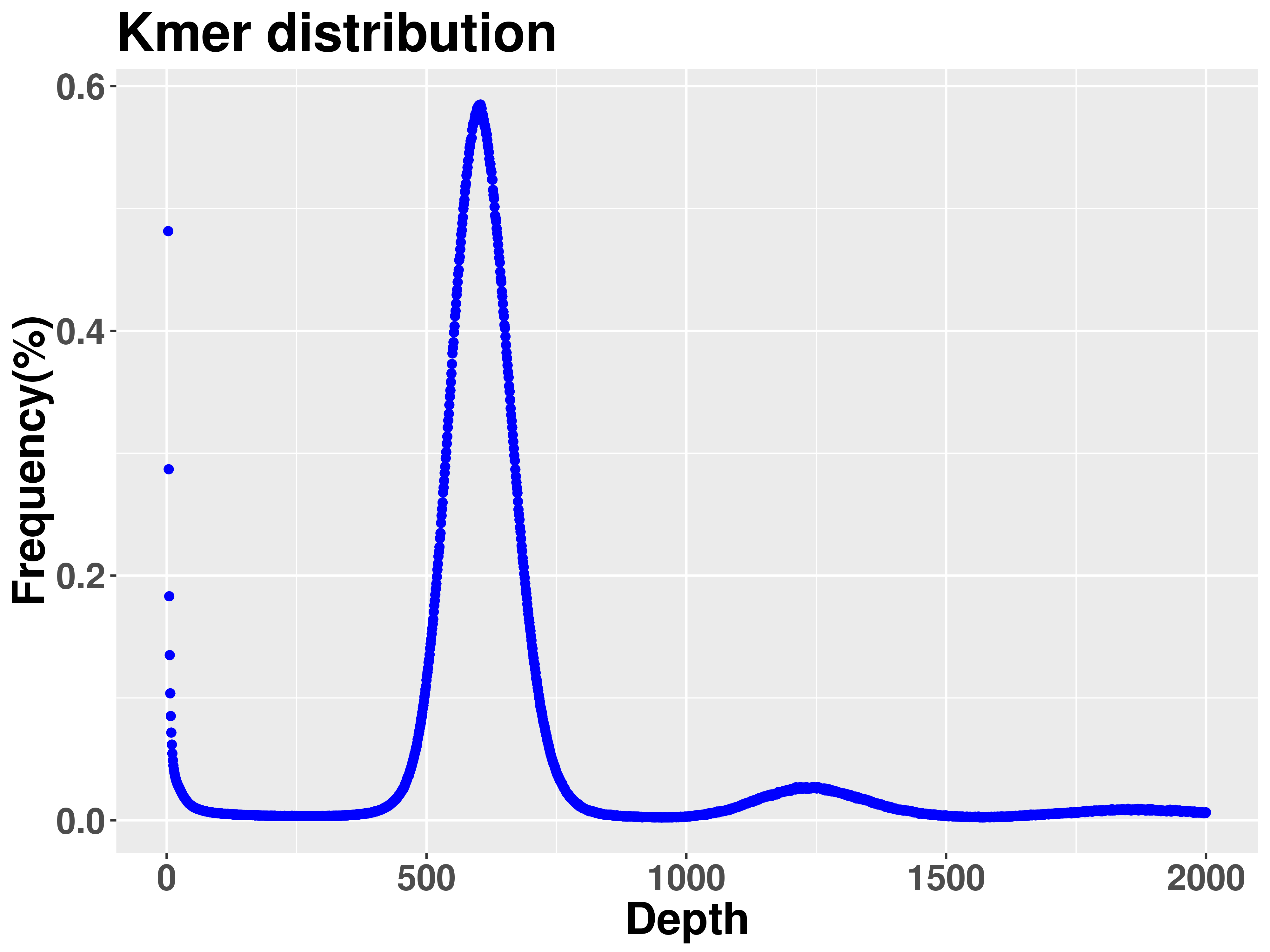

Supplement: Supplementary file 1 [file genes-13-01628-s001.zip › Figure S1.jpg]

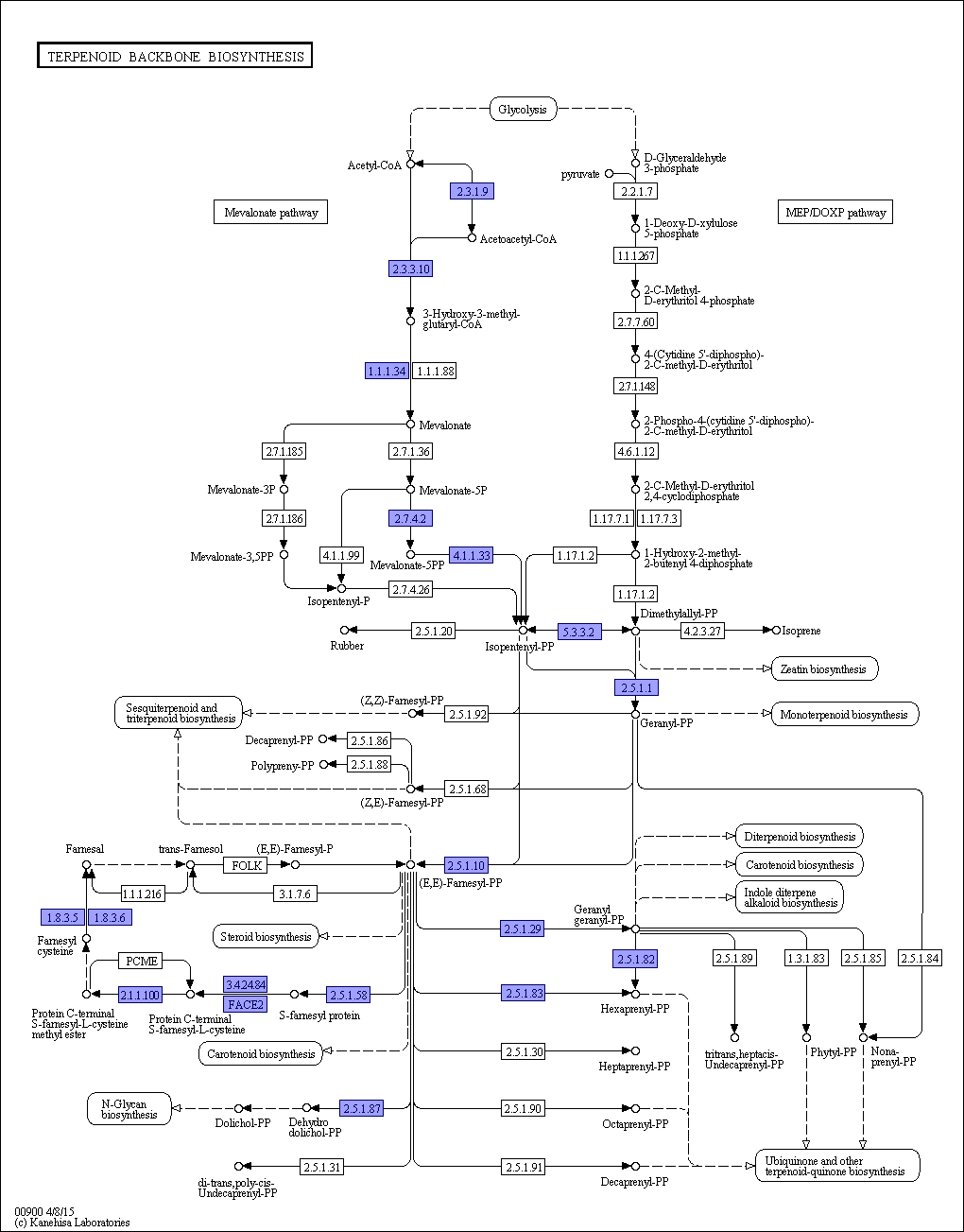

Supplement: Supplementary file 1 [file genes-13-01628-s001.zip › Figure S2.png]
